# TB prevention activities in the WHO European Region

**DOI:** 10.5588/ijtldopen.24.0350

**Published:** 2024-08-01

**Authors:** A. Matteelli, Z. Mkrtchyan, T. Masini, A. Hovhannesyan, G. Kuchukhidze, S. Ahmedov, P. Kerndt, L. Rossi, A. Yedilbayev, D. Falzon, A. Dadu

**Affiliations:** ^1^ Institute of Infectious and Tropical Diseases, WHO Collaborating Centre for Tuberculosis Prevention, University of Brescia, Brescia, Italy;; ^2^ London School of Hygiene & Tropical Medicine, London, UK;; ^3^ Independent Consultant, Lucca, Italy;; ^4^ Regional Office for Europe, WHO, Copenhagen, Denmark;; ^5^ United States Agency for International Development, Washington DC, USA;; ^6^ Global Tuberculosis Programme, WHO, Geneva, Switzerland

**Keywords:** tuberculosis, TPT, TB preventive treatment, TB screening

## Abstract

**BACKGROUND:**

TB preventive treatment (TPT) is the primary available healthcare intervention to reduce the risk of progression from TB infection to TB disease. The WHO Regional Office for Europe established the European Prevention and Systematic Screening Initiative to End TB (PASS) to scale up activities related to the programmatic management of TPT. In the absence of a system to measure and monitor preventive activities, a baseline assessment survey was carried out to provide a reference to monitor the scale-up of the intervention.

**METHODS:**

This was a semi-structured survey including 52 questions that was developed, implemented in the WHO-hosted LimeSurvey data form and sent to focal points in the 55 countries and territories in the European Region between September and October 2023. The questions covered TPT, systematic screening and infection prevention and control.

**RESULTS:**

A total of 28 questionnaires were returned, corresponding to an overall 51% response rate. Most national policies for TPT and TB screening are in line with the latest WHO guidelines. However, implementation of TB screening, prevention, and infection control activities is lagging. Results are presented separately for high-priority and low-priority countries.

**CONCLUSION:**

The survey identified several important areas that the PASS initiative will focus on to accelerate efforts towards reaching the targets set at the 2027 UN High-Level Meeting on TB for preventive therapy in the European Region. This will require a massive scale-up of efforts and larger investments, as well as coordinated approaches and interventions across the ‘cascade’ of prevention, from the identification of target populations to the completion of treatment.

## RÉSUMÉ

**CONTEXTE:**

Le traitement préventif de la TB (TPT) est une intervention majeure en santé publique pour réduire le risque de progression de l'infection TB vers la maladie TB. L'initiative européenne de prévention et de dépistage systématique pour mettre fin à la TB (PASS) a été lancée par le bureau régional de l'OMS pour l'Europe afin de renforcer les activités liées à la gestion programmatique du traitement préventif de la TB. Une enquête d'évaluation de base a été menée en l'absence d'un système de mesure et de suivi des activités de prévention, afin de fournir une référence pour suivre l'intensification de l'intervention.

**MÉTHODES:**

Il s'agit d'une enquête semi-structurée composée de 52 questions, conçue, mise en place dans le formulaire de données LimeSurvey hébergé par l'OMS, et envoyée aux points focaux dans les 55 pays et territoires de la Région européenne entre septembre et octobre 2023. Les questions abordaient le TPT, le dépistage systématique, ainsi que la prévention et le contrôle des infections.

**RÉSULTATS:**

Au total, 28 questionnaires ont été retournés, ce qui équivaut à un taux de réponse global de 51%. La plupart des politiques nationales en matière de TPT et de dépistage de la TB sont conformes aux dernières directives de l'OMS. Cependant, la mise en œuvre des activités de dépistage de la TB, de prévention et de lutte contre l'infection est en retard. Les résultats de l'initiative PASS sont présentés de manière distincte pour les pays hautement prioritaires et les pays faiblement prioritaires.

**CONCLUSION:**

L'enquête a révélé plusieurs domaines clés sur lesquels l'initiative PASS se concentrera pour accélérer les efforts visant à atteindre les objectifs de 2027 de l'UN High-Level Meeting en matière de traitement préventif dans la Région européenne. Cela exigera une intensification massive des efforts et des investissements accrus, ainsi que des approches et des interventions coordonnées tout au long de la « cascade » de la prévention, depuis l'identification des populations cibles jusqu'à l'achèvement du traitement.

The provision of TB preventive treatment (TPT) is the main available healthcare intervention to reduce the risk of progression from TB infection (TBI) to TB disease.^1^ The programmatic management of TPT (PMTPT) is critical for populations at high risk of progression to disease, including people with HIV and contacts of bacteriologically confirmed pulmonary TB patients. This intervention plays a key role within the larger framework of preventive actions envisaged under Pillars 1 and 2 of the WHO End TB Strategy.^2,3^

The WHO European Region (EURO) is in an ideal position to promote and support the PMTPT strategy as it has the lowest estimated TB incidence rate among all WHO regions, at 21/100,000 population, with many countries progressing towards pre-elimination.^4,5^ At the first United Nations High-Level Meeting (UNHLM) on TB, global targets were set for 2018 to 2022, calling for 2 million people receiving TPT in the European Region.^6,7^ However, TPT roll-out in the Region remained low, falling significantly behind the committed target. Indeed, only 667,000 people received TPT in that period.^8^ While 93% of the target was reached among household contacts aged under 5 years, only 44% of older contacts and 12% of people with HIV started TPT.

At the second UNHLM-TB held in September 2023, even more ambitious global TPT targets were set, including the provision of TPT to 45 million people between 2023 and 2027, including 15 million people with HIV and 30 million contacts of all ages.^9^ To support scaling up TPT-related interventions and contribute to reaching these ambitious targets, EURO presented the revised TB Action Plan for the WHO European Region for 2023–2030,^10^ which was endorsed by all member states at the 72^nd^ session of the Regional Committee for Europe in 2022. The plan aims to end the TB epidemic in the Region by 2030 and includes enhancing TB screening and providing TPT to 1.35 million people living with HIV and 200,000 household contacts aged under 5 years.

Within this framework, EURO established the initiative ‘PASS to end TB in Europe: accelerated efforts on Prevention and Systematic Screening to End TB in the WHO European Region by 2030’.^11^ The initiative, which focuses on 18 high-priority countries (HPCs),^*^ intends to support the implementation of services for prevention and screening by providing support on policy adaptation and implementation, capacity building and enhancing monitoring and evaluation. The initiative will target prioritised countries and will be based on the collaboration among focal points at national TB programmes (NTPs) supported by WHO consultants and with the active engagement of civil societies and affected communities.

The initial step of the PASS initiative was a baseline cross-sectional multi-country survey in the European Region to assess current national policies and practices along the TB preventive pathway. Preliminary results were presented and discussed with country representatives at an in-person meeting in Istanbul, Turkey, in October 2023 and used to design targeted, country-specific interventions.^12^

Here, we highlight health system challenges to the implementation of a PMTPT strategy as they emerged from the survey and the meeting discussions. We also describe how the PASS initiative can accelerate interventions toward TB elimination in the European Region.

## METHODS

A semi-structured survey comprising 52 questions was developed and implemented in the WHO-hosted LimeSurvey data form. The questions covered three main thematic areas in TB prevention, namely TPT, systematic screening and infection prevention and control (IPC) and aimed to assess the coverage of national policies on eligibility for TPT and systematic screening, as well as the availability of protocols, practices and other resources to facilitate the implementation of TPT, IPC and systematic screening activities. The questions were adapted from a questionnaire used in the European Region in 2014 and in 2021 and enhanced with new variables from a similar study conducted in mid-2023 by the WHO Western Pacific Regional Office (manuscript in preparation) to ensure a consistent approach across studies in such a way that findings could be compared across regions at a later stage.

The survey was developed in English and translated into Russian. Between September and October 2023, an invitation to participate in the survey was sent to NTP managers and TB surveillance and response monitoring focal points in the 55 countries and territories in the European Region. Participants were asked to answer an online questionnaire or print and mail the completed questionnaire to the study investigators. Data from the questionnaires completed offline were manually entered into LimeSurvey, and all questionnaires were analysed together.

Responses received were exported from LimeSurvey to R for analysis.^13^ Descriptive statistics (frequencies, proportions, means, medians) were calculated for all countries and grouped into HPCs – that is, the countries that the PASS initiative aims to target – and low-priority countries (LPCs).^14^

Additional information was collected through in-person discussions during a meeting convened in Istanbul from 30 October to 2 November 2023, during which 13 out of 16 participating NTP managers from HPCs met with study investigators. The questionnaires had variable completion rates across respondents. Therefore, results are reported using the number of responses for each specific question as a denominator. Only percentages of countries that provided a certain response are given for multiple-choice questions.

All the respondents were representatives of NTPs and included programme coordinators, TB experts, and heads of TB clinics, all of whom were nominated by respective Ministries of Health.

Ethical approval was not required for this survey, as it did not involve human subjects.

## RESULTS

A total of 28 questionnaires were returned, corresponding to an overall 51% response rate, including 17 from HPCs (94% response rate) and 11 from LPCs (30% response rate). National guidelines on TPT, IPC and systematic screening were available in 15 of the 16 HPCs (94%). Most countries (9/13, 70%) updated their guidelines on TPT and systematic screening in 2022 or 2023, while only 4/11 (36%) reported that national IPC guidelines were updated in the prior two years. Respondents from the majority of the surveyed HPCs indicated that implementation of TPT, IPC and systematic screening activities are as ‘good, but need improvement’ (12/16, 75%; 9/15, 60%; 10/16, 63%), while the implementation of TPT activities was perceived to be ‘good’ in most LPCs (6/11, 55%).

**Figure 1.**
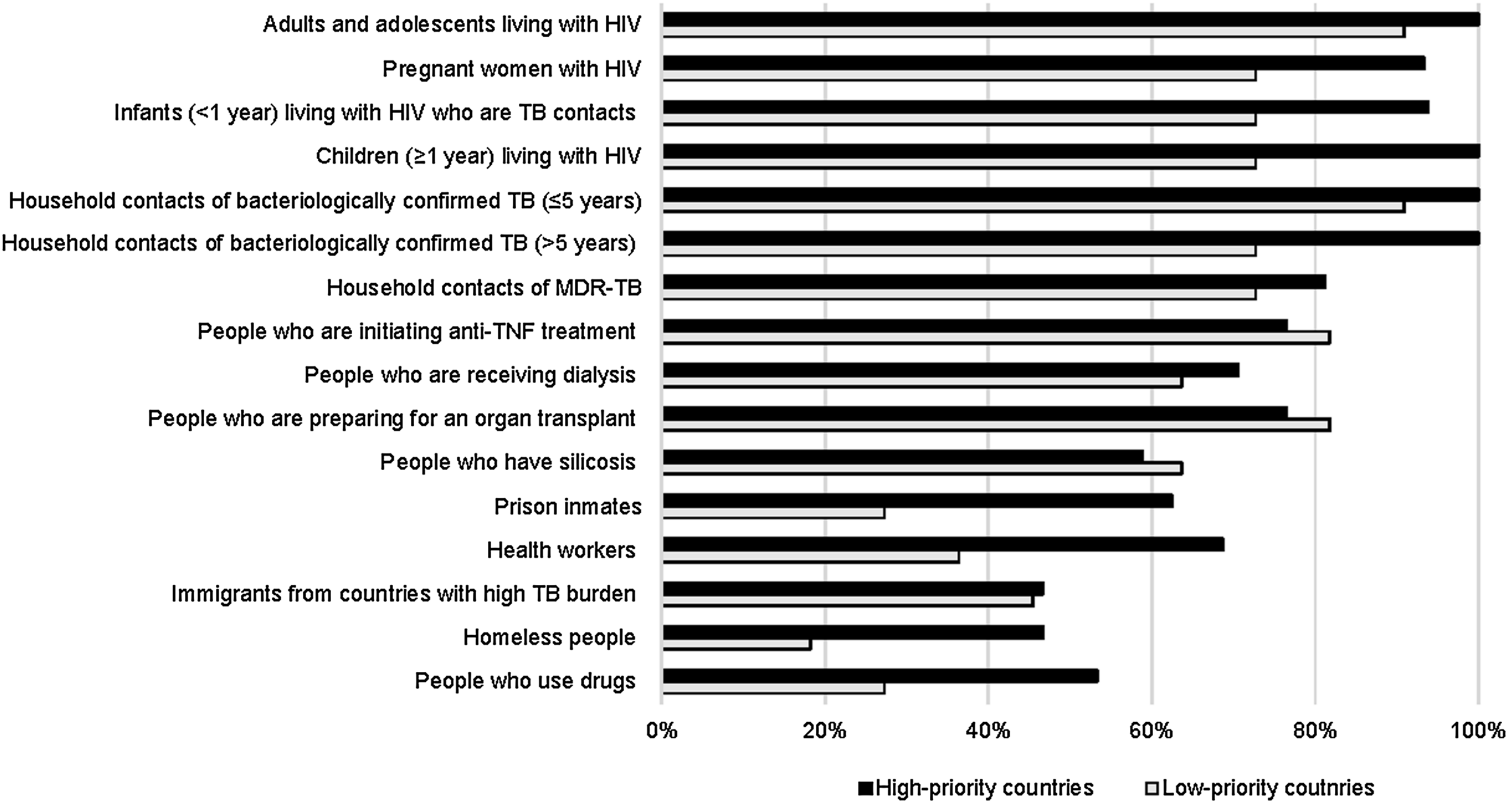
TPT eligibility for at-risk populations in high-priority and low-priority countries. MDR-TB = multidrug-resistant TB; TNF = tumour necrosis factor; TPT = TB preventive therapy.

All HPCs indicated that people living with HIV and household contacts (of all ages) of bacteriologically confirmed TB patients were eligible for TPT. TPT eligibility for other at-risk populations varied, with immigrants from countries with a high TB burden and homeless people being eligible for TPT in 47% of HPC countries. LPCs reported similar results; however, a lower proportion of LPCs indicated TPT eligibility for healthcare workers (36%), prisoners (27%), people who use drugs (27%) and homeless people (18%) compared to HPCs (Figure 1).

In most countries (88% HPCs, 100% LPCs), a test for TB infection was required before initiating TPT. The TB skin test (TST) and IGRA (QuantiFERON, Qiagen, Hilden, Germany; or T-SPOT.*TB*, Oxford Immunotec, Abingdon, UK) were used in 88–100% of the countries. A chest X-ray was required before TPT initiation in all surveyed countries (Supplementary Table S1).

**Figure 2.**
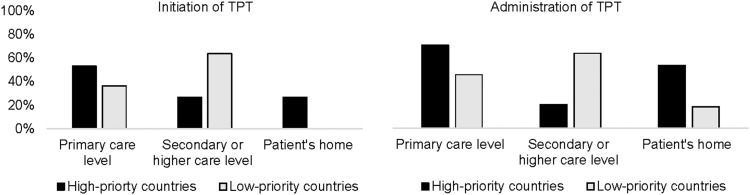
Initiation and administration of TPT in high-priority and low-priority countries. TPT = TB preventive treatment.

In HPCs, TPT initiation occurred mainly at the primary healthcare level (53%) and in TB clinics (47%), while case management during treatment, including medicine refills, was primarily a responsibility of primary healthcare (71%). TPT delivery at the patient's home was reported by 53% of the countries, and only 12% of the countries indicated that administration was also done at community-based facilities. Results differed for LPCs, where secondary-level facilities played a major role in treatment initiation (done at TB clinics for 73% of the countries) and treatment administration (done at secondary-level facilities for 64% of the countries) (Figure 2).

**Figure 3.**
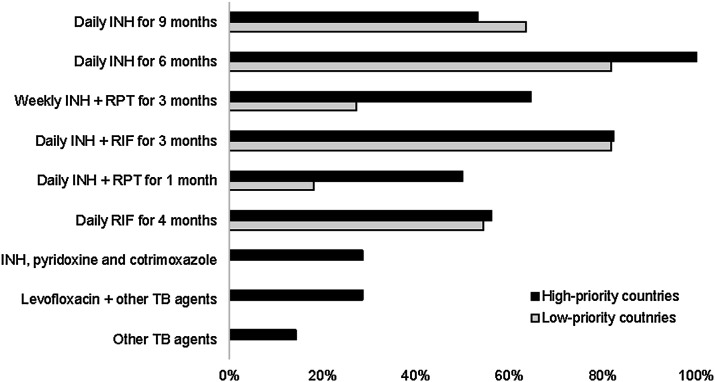
TPT regimens are adopted in high-priority countries and low-priority countries. INH = isoniazid; RPT = rifapentine; RIF = rifampicin; TPT = TB preventive treatment.

In most HPCs, national guidelines included 6-month isoniazid and 3 months isoniazid and rifampicin (3HR) as recommended regimens (100% and 82%, respectively). In addition, guidelines in more than half of HPCs included a 3-month regimen of weekly isoniazid and rifapentine (3HP) and one month of daily isoniazid and rifampicin (1HP) (65% and 50%, respectively) as well as a regimen of 4 months of rifampicin monotherapy (4R) (56%). A similar trend was noted for LPCs. However, the use of 3HP and 1HP was lower in LPCs (27% and 18% of LPCs reported their use, respectively) (Figure 3).

TPT for household contacts of bacteriologically confirmed multidrug-resistant TB (MDR-TB) cases was recommended in 81% (13/16) of HPC. However, only 31% (4/13) indicated that levofloxacin (LVX) based regimens were used. None of the LPCs indicated LVX-based regimens as an option for MDR-TB contacts in national guidelines, even though 73% of them recognised their potential eligibility for the regimen.

Approaches for treatment monitoring and follow-up of TPT were absent or not standardised. Monthly treatment monitoring was indicated by 29% of HPCs, but some also reported a higher frequency of treatment monitoring (daily: 6%, weekly: 18%), and 41% reported ad hoc approaches based on the type of TPT regimen administered, or on the expected client compliance. Visits to health facilities that initiated TPT and phone calls to healthcare workers were used mainly by people on TPT to report adverse drug events in HPCs (94% and 76%, respectively) and LPCs (100% and 82%, respectively). The use of smartphone applications to report adverse events was indicated by 44% of HPCs but by no LPC. All countries recommended that a blood test be done in case of side effects during the administration of TPT (Supplementary Table S2).

Target groups for systematic screening prioritised by surveyed HPCs include people living with HIV (94%), people in penitentiary institutions (94%), people with an untreated fibrotic lesion seen on chest X-ray (94%), household contacts and other close contacts of individuals with TB disease (88%), refugees (81%) and migrants (75%). A few other groups were also reported, such as communities in remote or isolated areas (21%), the general population in areas with an estimated TB prevalence of 0.5% or higher (20%), urban poor communities (14%) and indigenous populations (7%). In general, a lower proportion of LPCs reported systematic screening in at-risk populations (e.g., people with malnourishment, pregnant women, smokers, people with chronic lung diseases, people with diabetes, people with chronic lung disease and other vulnerable or marginalised groups with limited access to healthcare) (Supplementary Figure S1).

Screening for TB disease was done mainly by chest X-ray and symptom check in both HPCs and LPCs (chest X-ray: 100% HPCs and 91% LPCs; symptom check: 88% HPCs and 73% LPCs). WHO four-symptom screening tool was used much more in HPCs (67%) than in LPCs (18%). Molecular WHO-recommended rapid diagnostic tests were reported by 60% of HPCs and 45% of LPCs. In most HPC countries, systematic screening was implemented in health facilities (75%) or mobile units (73%), while in LPCs, it was mainly implemented in health facilities (55%). Implementation in community-based facilities is low irrespective of the setting (HPCs: 27%; LPCs: 9%).

The majority of HPCs (67%) reported using computer-aided detection (CAD), in contrast to most LPCs (91%) that did not report using it. CAD is implemented at various healthcare levels in HPCs, mostly in public facilities (only 20% in private clinics), where the service is primarily financed by external funding (53%).

Most of the surveyed countries reported adopting a variety of IPC interventions. Almost all countries (93% HPCs and 100% LPCs) reported having IPC committees in health facilities, with specific staff designated to oversee TB IPC interventions. The use of particulate respirators (N95, FFP2) in settings with a high risk of transmission was widely spread across HPCs (94%) and LPCs (100%). TB screening and provision of TPT were offered to healthcare workers in all HPCs surveyed, compared to 10% of LPCs (Supplementary Table S3).

## DISCUSSION

The WHO Regional Office for Europe engaged in scaling up TB preventive interventions in the region by launching the PASS project, in partnership with other partners and stakeholders, in the frame of the elimination strategy. In the absence of a system to measure and monitor preventive activities, a baseline assessment survey was carried out to provide a reference to monitor the scale-up of the intervention.

Our survey results confirm the importance of WHO guidelines to prompt adaptation and adoption of countries’ policies in TB screening and TPT. On the other hand, updated IPC country guidelines are still delayed, as WHO just published new recommendations in this area.^15^ Importantly, there is a clear gap between policy adaptation and implementation of TPT, IPC and systematic screening interventions. Coherently, the main role of the PASS initiative is the translation of policies into actions.

People living with HIV and household contacts of people with bacteriologically confirmed TB are target populations for PMTPT in all countries, although coverage is difficult to measure and may vary by country. Migrants from high TB burden settings represent another important target population in the region. While WHO and the European Centre for Disease Control do not recommend universal TB and TBI testing of refugees arriving in European countries from Ukraine, testing may be offered to certain individuals when the risk for TB is heightened.^16^ By June 2023, it is estimated that over 5.9 million people fled Ukraine as refugees and asylum seekers, with Poland hosting 976,812 (16.5%) of them.^17^ Host countries such as Poland and Czechia had to provide treatment to a very large number of TB patients from Ukraine, many of whom had MDR-TB disease, resulting in extremely burdened health services.^18,19^

WHO recommends that a lead-in test is not required before initiating TPT in high-risk populations (people living with HIV and children under 5 years of age).^1^ However, with TBI prevalence among adult household contacts estimated to be about 50% in the European Region,^20^ TBI testing is an important intervention from a health system perspective as it reduces the number of people who are given TPT, thus optimising resources. Restricting TPT provision also has the major advantage of avoiding unnecessary risks of toxicity for people who do not have TBI. The risk of overtreatment also raises ethical issues and tensions that should not be overlooked.^21^ Indeed, all responding countries indicated the availability of TST and IGRAs. Countries are also starting to use the new category of WHO-recommended skin tests based on specific *M. tuberculosis* antigens, early secreted antigenic target 6 and culture filtrate protein 10,^22^ particularly Diaskin (Diaskintest;^®^ Generium Pharmaceuticals, Volginsky Settlement, Russia) (47%) and CyTB (Mylab Discovery Solutions, Pune, India) (7%). While not yet in widespread use, the performance of antigen-based skin tests is comparable to IGRA and is less costly. Therefore, they could be a viable testing option before TPT enrolment for countries in the region. Nonetheless, if TB testing is not available, contacts who are at risk of TB should not be deprived of TPT. Further operational research is needed to establish TBI prevalence, identify risk factors for infection and inform policy for offering TPT to contacts when testing is not available.

The region's wide availability of radiological equipment can support screening activities (to rule in active disease) and TPT provision (to rule out active disease). However, access within country contexts is uncertain. Most countries are aware of the development of mobile digital X-ray with CAD and distance reading technologies, which are used in some programmes. Obstacles and enablers for increased uptake of this technology should be investigated as a key tool to accelerate PMTPT interventions.

The implementation of WHO-recommended shorter TPT regimens is an essential intervention to optimise PMTPT as these are associated with better adherence and higher treatment completion rates. Although isoniazid is still an option for TPT in most countries, shorter TPT regimens are usedtoo, especially 3HR. In LPCs, rifapentine-based regimens such as 3HP and 1HP are overall much less implemented, presumably because of the lack of availability of rifapentine in European Union countries.^23^ Indeed, rifapentine has not been approved yet by the European Medicines Agency, hindering EU countries’ access to the regimens containing the drug.^24^ Supporting countries in moving towards shorter TPT regimens and discontinuing IPT should be prioritised as one of the key PMTPT interventions, considering that suitable formulations for all regimens are now available across the age spectrum (including for children).

Given the high MDR-TB burden in the WHO European Region, effective treatment of exposed contacts would prevent MDR-TB development and ultimately reduce its burden and health system costs. Even though most surveyed countries indicated that household contacts of MDR-TB cases are eligible for TPT, the vast majority did not indicate the use of LVX-based regimens. The upcoming WHO’s updates to TPT guidelines,^25^ where the LVX-based regimen for TPT will receive a strong recommendation based on the results from two landmark trials,^26,27^ should facilitate a rapid change in the management of contacts of people with MDR-TB. Contacts to DR-TB should be prioritised for TBI diagnostic testing, and negative contacts should be retested 10–12 weeks after exposure ends to ensure any transmission is identified and treated. This is another area where the PASS initiative could enable rapid scale-up.

Historically, TPT initiation and treatment monitoring were done in TB clinics, with an adverse impact on adherence and added costs for people who needed to travel longer distances to reach these facilities. Promisingly, most HPCs surveyed initiate and provide administration of TPT at primary healthcare facilities, signalling an important shift toward better models for TPT delivery. However, the potential of community-based facilities has not yet been fully exploited. Despite recognising the importance of mobilising and partnering with communities to improve the reach, awareness, acceptability, and sustainability of PMTPT interventions as an essential strategy to generate demand for TPT, only a minority of the surveyed HPCs indicated that TPT initiation is done at community-based facilities. Patient-centred approaches (administration of TPT at the patient’s home) were also demonstrated by more than half of the surveyed HPCs. In general, moving away from facility-based approaches to household-based/family-centred approaches with community-based treatment support can facilitate the implementation of TPT while also offering the opportunity to promote the integration of TB and HIV screening at the community level. Because there is no universally applicable model, more research is needed to establish the appropriate ways for community engagement in countries with different TB epidemiological profiles and healthcare infrastructures. The role of the PASS initiative will be key to design country-specific interventions in the 18 HPCs included in the project.

Smartphone technologies and applications to promote spontaneous reporting of adverse drug reactions can be a precious resource for treatment monitoring.^28^ Experience gained during the COVID pandemic on video-supported treatment for TB could easily be adapted to the management of side effects of preventive therapy.^29^ Almost half of surveyed HPCs mentioned the use of smartphone applications for this scope, which sounds very promising.

## CONCLUSIONS

Reaching the 2027 UNHLM targets for TPT provision will require a massive scale-up of efforts and larger investments, as well as coordinated approaches and interventions across the “cascade” of prevention, from the identification of target populations to the completion of treatment. Country- and region-specific interventions should be context-specific and designed based on epidemiological assessment and recognition of strengths and gaps. The baseline assessment conducted as part of the PASS initiative provides useful insights into current challenges, gaps, and opportunities that can be exploited to streamline efforts to achieve regional targets.

## Supplementary Material



## Footnotes

* The 18 high-priority countries for TB control in WHO European Region are Armenia, Azerbaijan, Belarus, Bulgaria, Estonia, Georgia, Kazakhstan, Kyrgyzstan, Latvia, Lithuania, Republic of Moldova, Romania, Russian Federation, Tajikistan, Turkey, Turkmenistan, Ukraine and Uzbekistan.
