# Supplementary material for: TB prevention activities in the WHO European Region
Source: IJTLD Open. 2024 Aug 1;1(8):349–54. doi: 10.5588/ijtldopen.24.0350 (PMC11308406; doi:10.5588/ijtldopen.24.0350)
Supplement: Supplementary file 1 [file ijtldopen0350_supplementarydata1.pdf]

Supplementary material

A landscape of tuberculosis prevention activities in the World Health Organization European Region

Supplementary table 1. Tests and examinations before initiation of TPT

	High-priority countries	Low-priority countries	All countries
**Chest Xray required**	100%	100%	100%
**Test for TB infection required**	88%	100%	93%
**Tests used**			
**TB skin test: C-TST or ESAT6-CFP10**	7%	0%	4%
**TB skin test: Diaskintest**	47%	0%	29%
**Tuberculin skin test**	100%	100%	100%
**IGRA: T-SPOT**	20%	55%	35%

Supplementary table 2. Monitoring of TPT reporting of adverse events

**Frequency of TPT monitoring**	**High-priority countries**	**Low-priority countries**	**All countries**
Daily	6%	0%	4%
Weekly	18%	0%	11%
Monthly	29%	82%	50%
Quarterly	6%	0%	4%
At the end of the treatment period	0%	9%	4%
Other	41%	9%	29%
**Monitoring of adverse events by healthcare providers**			
Phone call at a regular interval	65%	55%	61%
Home visits by staff from the health facility that administered TPT	59%	18%	43%
Home visits by other health facility staff	21%	0%	12%
Through community outreach workers	40%	9%	27%
Smartphone application	38%	0%	22%
**Reporting of adverse events by patients**			
Phone call to health workers	76%	82%	79%
Visit health facilities that initiated TPT	94%	100%	96%
Visit other health facilities (e.g. general practitioners or other clinics or hospitals)	56%	45%	52%
Smartphone applications	44%	0%	26%

Supplementary Figure S1. Groups eligible for systematic screening

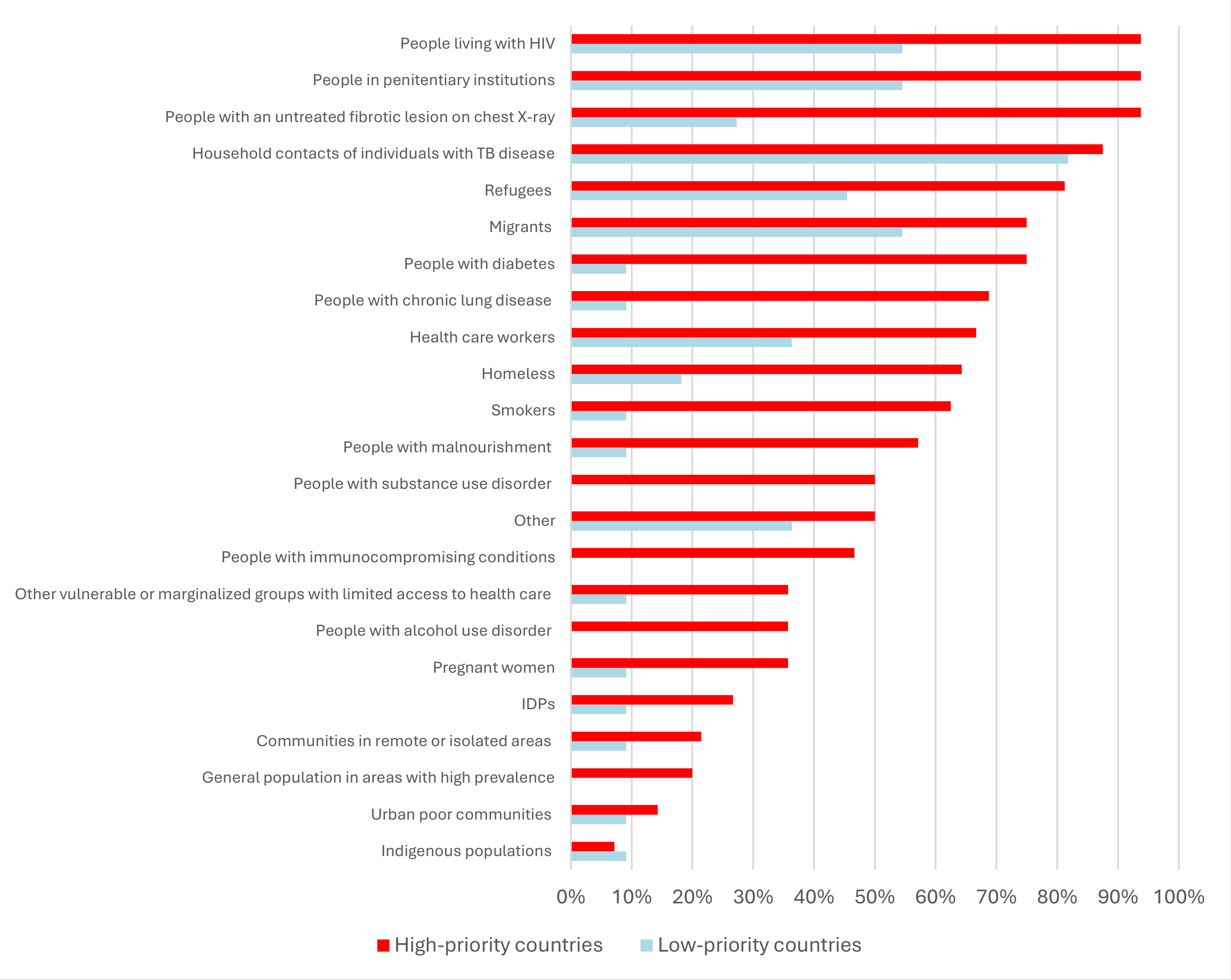

Supplementary Table S3. TB infection prevention and control activities

Supplementary table 4 TB Infection Prevention and Control Activities	**High-priority countries**	**Low-priority countries**	**All countries**
Annual screening for healthcare workers	100%	10%	65%
IPC risk assessments for high-risk settings	70%	67%	69%
Upper-room germicidal ultraviolet systems implemented	100%	40%	78%
Healthcare staff sensitized on airborne IPC	93%	82%	88%
Ventilation systems implemented	100%	78%	92%
Respiratory protection equipment included in procurement plans	100%	80%	95%
Staff to monitor of TB IPC activities	100%	89%	95%
IPC committees established	93%	100%	96%
Triage of people with TB symptoms	100%	91%	96%
Particulate respirators (N95, FFP2) are implemented	94%	100%	96%
Respiratory separation/isolation done	100%	100%	100%
Prompt initiation of treatment	100%	100%	100%
Respiratory hygiene implemented	100%	100%	100%
IPC mandate includes airborne infection and TB	100%	100%	100%
